# Performance evaluation of the cobas SARS-CoV-2 Duo, a novel qualitative and quantitative assay, for the detection of SARS-CoV-2 RNA

**DOI:** 10.1128/spectrum.01369-23

**Published:** 2023-11-01

**Authors:** Yang-Di Su, Chih-Cheng Lai, Tsai-Hsiu Lin, Wei-Cheng Chen, Po-Ren Hsueh

**Affiliations:** 1 Department of Laboratory Medicine, China Medical University Hospital, School of Medicine, China Medical University, Taichung, Taiwan; 2 Department of Internal Medicine, Division of Hospital Medicine, Chi Mei Medical Center, Tainan, Taiwan; 3 Department of Internal Medicine, Division of Pulmonary and Critical Care Medicine,China Medical University Hospital, Taichung, Taiwan; 4 Graduate Institute of Biomedical Sciences and School of Medicine, College of Medicine, China Medical University, Taichung, Taiwan; 5 Department of Internal Medicine, Division of Infectious Diseases, China Medical University Hospital, School of Medicine, China Medical University, Taichung, Taiwan; 6 PhD Program for Aging, School of Medicine, China Medical University, Taichung, Taiwan; London Health Sciences Centre, London, Ontario, Canada

**Keywords:** cobas SARS-CoV-2 Duo, COVID-19, qualitative, quantitative, SARS-CoV-2

## Abstract

Viral load quantification of severe acute respiratory syndrome coronavirus 2 (SARS-CoV-2) is a critical measure for monitoring clinical responses of patients with COVID-19. The cobas SARS-CoV-2 Duo (Roche Molecular Systems, Inc.) uses the cobas 6800/8800 platform and is an automated real-time reverse transcription PCR (RT-PCR) assay for the qualitative detection of SARS-CoV-2 RNA (ORF1a and ORF1a/b non-structural regions) in nasal and nasopharyngeal specimens. This assay also performs quantitation of the SARS-CoV-2 RNA level in the collected specimen. The precision, accuracy, and linearity of the cobas SARS-CoV-2 Duo test were assessed and correlated with the cycle threshold (Ct) value of the cobas SARS-CoV-2 assay, and the cobas Liat SARS-CoV-2 and influenza A/B assay (Inf A/B) (cobas Liat) in 201 clinical nasopharyngeal swab specimens. The analytical measurement range of the cobas SARS-CoV-2 Duo has been verified to be 1.0E + 02 IU/mL to 1.0E + 09 IU/mL. The between- and within-run precision evaluation and the linearity results (*R* ^2^ = 0.9961) of the cobas SARS-CoV-2 Duo were acceptable based on the verification criteria. A total of 201 nasopharyngeal specimens were evaluated. The positive, negative, and total agreements between the cobas SARS-CoV-2 Duo and the cobas SARS-CoV-2 assay were 93.5% (157 of 168), 75% (33 of 44), and 92.5% (186 of 201), respectively. The qualitative results (Ct values) from the cobas SARS-CoV-2 and the cobas Liat demonstrated good agreement with the quantitative results from the cobas SARS-CoV-2 Duo assay. In conclusion, the diagnostic performance of the cobas SARS-CoV-2 Duo assay is appropriate. It is a precise, accurate, and sensitive method for the detection of SARS-CoV-2 RNA and is comparable to two qualitative assays.

**IMPORTANCE:**

Quantitative SARS-CoV-2 tests for viral load are necessary to guide patient treatment, as well as to determine infection control measures and policies. Although the real-time RT-PCR assays can report the Ct value to estimate the viral load, there are several serious concerns regarding the use of Ct values. Importantly, Ct values can vary significantly among between- and within-run methods. The diagnostic performance of the cobas SARS-CoV-2 Duo is appropriate. It is a precise, accurate, and sensitive method for the detection of SARS-CoV-2 RNA and is comparable to two qualitative assays (the cobas SARS-CoV-2 and the Liat cobas SARS-CoV-2 and Inf A/B). In contrast, using the Ct value to estimate viral load is not reliable, and utilization of a quantitative detection test, such as the cobas SARS-CoV-2 Duo, to accurately measure the viral load is needed.

## INTRODUCTION

At the end of 2019, the coronavirus disease 2019 (COVID-19) outbreak, caused by a novel virus, the severe acute respiratory syndrome coronavirus 2 (SARS-CoV-2), occurred in Wuhan, China. The virus spread rapidly, resulting in a global pandemic (1 – 5). Within a very short time, many diagnostic modalities were developed to detect SARS-CoV-2 RNA (6). According to the World Health Organization (WHO), as of 20 January 2023, there have been 663,640,386 confirmed cases of COVID-19, including multiple waves with different variants around the world (2, 4, 5, 7 – 9). At present, most reported cases have been identified via the qualitative detection of SARS-CoV-2 RNA, antigens, or antibodies (10 – 19). Rapid antigen or antibody tests can provide an early diagnosis of SARS-CoV-2 infection, but they cannot provide accurate information on the viral load (20 – 23). In most clinical settings, qualitative assessments using rapid antigen or antibody tests are sufficient for identifying COVID-19 so that timely containment and mitigation measures may be initiated. However, there are instances when quantitative SARS-CoV-2 tests are necessary to guide patient treatment as well as determine infection control measures and policies.

Although real-time reverse transcription PCR (RT-PCR) assays can report the cycle threshold (Ct) value to estimate the viral load, there are several serious concerns regarding the use of Ct values (24). Importantly, Ct values can vary significantly among between- and within-run methods. The median Ct value of different Food and Drug Administration Emergency Use Authorization assays has been shown to vary by as much as 14 cycles (24). Therefore, a more accurate and standardized measurement of the viral load is urgently needed.

This study aimed to evaluate the performance of a new generation of standardized SARS-CoV-2 nucleic acid quantitative tests, specifically the cobas SARS-CoV-2 Duo assay (Roche Molecular Systems, Inc., Branchburg, NJ, USA). The evaluation involved three steps. First, the diagnostic performance of the cobas SARS-CoV-2 Duo was evaluated. Second, the Ct value of the cobas SARS-CoV-2 Duo results was correlated. Third, we evaluated the correlation between the viral load measurements of the cobas SARS-CoV-2 Duo with the Ct values of the cobas SARS-CoV-2 (Roche Molecular Systems, Inc.) and the cobas Liat SARS-CoV-2 and influenza A/B (Inf A/B) (cobas Liat, Roche Molecular Systems, Inc.) assays.

## MATERIALS AND METHODS

### cobas SARS-CoV-2 Duo

The cobas SARS-CoV-2 Duo assay is based on a fully automated method of sample preparation (nucleic acid extraction and purification), PCR amplification, and detection using the cobas 6800 automation system (25). This real-time RT-PCR test uses 400 µL of viral transport medium from a nasal, nasopharyngeal, and oropharyngeal swab specimen to perform the analysis. The test also uses three external controls: a high-titer positive control, a low-titer positive control, and a negative control (NC). The high positive control (HPC) and the low positive control (LPC) were made by diluting stock material with a titer traceable to the First WHO International Standard for SARS-CoV-2 RNA [National Institute for Biological Standards and Control (NIBSC) code 20/146]. Each cobas SARS-CoV-2 Duo kit lot was calibrated and traceable to the First WHO International Standard for SARS-CoV-2 RNA (NIBSC code 20/146).

Briefly, the master mix used in this study included deoxyuridine triphosphate instead of deoxythymidine triphosphate, which was incorporated into the newly synthesized DNA (amplicon). Uracil-N-glycosylase was also included in the master mix to destroy contaminating amplicons from previous PCR runs. Automated data management was performed using the cobas 6800 software, which assigned test results to each sample. Selective amplification of target nucleic acids from the sample used highly conserved regions of SARS-CoV-2 RNA located in the ORF1a and ORF1a/b non-structural regions. The viral load was quantified against a non-SARS-CoV-2 armored RNA quantitation standard (RNA-QS), which was simultaneously extracted for each specimen during sample preparation. The RNA-QS also functioned as an internal control for monitoring amplification efficiency and RNA extraction.

### Assessment of precision of cobas SARS-CoV-2 Duo

The precision of the cobas SARS-CoV-2 Duo assay was evaluated through between-run and within-run tests, respectively. The negative samples were obtained from the cobas Buffer Negative Control Kit, and the high- and low-titer positive samples were from the cobas SARS-CoV-2 Duo Control kit. In the between-run tests, negative and two-level positive samples were measured in three independent batch runs. In the within-run tests, negative and two-level positive samples were measured in triplicates per batch run. The acceptance criterion of the negative sample was “targeted but not detected.” The acceptance criterion of the positive sample was “less than or equal to the twofold standard deviation, which was 0.10.”

### Assessment of the accuracy of cobas SARS-CoV-2 Duo

The accuracy of the cobas SARS-CoV-2 Duo was assessed according to the WHO International Standard for SARS-CoV-2 Virus (NIBSC code 20/146). Based on the NIBSC code 20/146 instructions, the final concentration of the preparation should be 7.70 log_10_ IU/mL after reconstitution in 0.5 mL of molecular-grade water. The acceptance range was 7.70 ± 0.3 log_10_.

### Assessment of linearity of cobas SARS-CoV-2 Duo

The analytical measuring range (AMR) of the cobas SARS-CoV-2 Duo was set at 1.0E + 02 IU/mL to 1.0E + 09 IU/mL. Linearity was assessed by a pooling sample mixed with patient nasopharyngeal swab specimens with a high-viral load tier (1.0E + 08 ~ 1.0E + 09 IU/mL). Tenfold serial dilutions of the samples in a negative simulated clinical matrix stabilized in viral transport media were made from the pooling sample to give an evaluation of linearity of the cobas SARS-CoV-2 Duo assay. Each of the testing samples was duplicated, and the acceptance criterion was the coefficient of determination, specifically, *R* ^2^ of >0.95.

### cobas SARS-CoV-2 assay

The cobas SARS-CoV-2 qualitative assay is based on fully automated sample preparation (nucleic acid extraction and purification), PCR amplification, and detection, using the cobas 6800 automation system (Roche Diagnostics) (26). In this study, 400 µL of viral transport medium from a nasal, nasopharyngeal, and oropharyngeal swab specimen was used for each real-time RT-PCR test. Selective amplification of the target nucleic acids from the sample in the ORF1/a non-structural region gene designated as target 1 (unique to SARS-CoV-2) and the structural protein envelope E gene as target 2 for the detection of pan-sarbecovirus, including SARS-CoV-2 virus. An internal RNA control was simultaneously extracted for each sample to control for the amplification efficiency and RNA extraction from the sample (26).

### cobas Liat assay

The cobas Liat assay using the cobas Liat system (Roche Diagnostics) is an automated multiplex real-time RT-PCR assay aimed for the rapid (approximately 20 minutes) qualitative detection and differentiation of SARS-CoV-2, influenza A, and influenza B virus RNA (27). In this study, 200 µL of viral transport medium from a nasal, nasopharyngeal, and oropharyngeal swab specimen was used for each analysis. When either or both target genes (ORF1a/b and N genes) of SARS-CoV-2 were detected, the cobas Liat assay assigned the specimen as positive. An internal process control (IPC) was also included. The IPC was present to control for adequate processing of the target virus through steps of sample purification and nucleic acid amplification, as well as to monitor the presence of inhibitors in the real-time RT-PCR processes.

## RESULTS

### Precision of cobas SARS-CoV-2 Duo

The between- and within-run precision tests were conducted using cobas external controls, including an LPC, an HPC and an NC. In the between-run precision tests, the mean ± standard deviation (SD) of LPC and HPC viral load results were 2.78 ± 0.03 and 6.82 ± 0.03 log_10_ IU/mL, respectively. The negative controls of the three batch runs were all targeted but not detected (Table 1). In the within-run precision tests, the mean ± SD of LPC and HPC viral load results were 2.71 ± 0.03 and 6.85 ± 0.02 log_10_ IU/mL, respectively. The triplicated negative controls were also all targeted but not detected in the same runs (Table 2). The total SD results (all 0.03) of the low and high positive controls in the between- and within-run tests were all acceptable. The results were less than 2 SDs of 0.1, as recommended by the manufacturer (25) (Tables 1 and 2).

**TABLE 1:** Assessment of precision of cobas SARS-CoV-2 Duo between run

Sample type/batch ID	LPC^*a*^		HPC^*a*^		NC^*a*^
	SARS-CoV-2 concentration (IU/mL)	SARS-CoV-2 concentration (log_10_ IU/mL)	SARS-CoV-2 concentration (IU/mL)	SARS-CoV-2 concentration (log_10_ IU/mL)	SARS-CoV-2 concentration (log_10_ IU/mL)
8502	5.76E + 02	2.76	6.67E + 06	6.82	TND^b^
8516	5.60E + 02	2.75	7.03E + 06	6.85	TND
8530	6.60E + 02	2.82	6.06E + 06	6.78	TND
Precision					
Sample type	LPC		HPC		
	IU/mL	Log_10_ IU/mL	IU/mL	Log_10_ IU/mL	
Mean	5.99E + 02	2.78	6.59E + 06	6.82	
Total SD^c^	4.39E + 01	0.03	4.00E + 05	0.03	

^*a*^ HPC, high positive control; LPC, low positive control; NC, negative control.

^*b*^ TND, target not detected.

^*c*^ SD, standard deviation.

**TABLE 2:** Assessment of precision of cobas SARS-CoV-2 Duo within run

Sample type/batch ID	LPC^*a*^		HPC^*a*^		NC^*a*^
	SARS-CoV-2 concentration (IU/mL)	SARS-CoV-2 concentration (log_10_ IU/mL)	SARS-CoV-2 concentration (IU/mL)	SARS-CoV-2 concentration (log_10_ IU/mL)	SARS-CoV-2 concentration (log_10_ IU/mL)
8502	5.76E + 02	2.76	6.67E + 06	6.82	TND^*b*^
8516	5.60E + 02	2.75	7.03E + 06	6.85	TND
8530	6.60E + 02	2.82	6.06E + 06	6.78	TND
Precision					
Sample type	LPC		HPC		
	IU/mL	Log_10_ IU/mL	IU/mL	Log_10_ IU/mL	
Mean	5.99E + 02	2.78	6.59E + 06	6.82	
Total SD^c^	4.39E + 01	0.03	4.00E + 05	0.03	

^*a*^ HPC, high positive control; LPC, low positive control; NC, negative control.

^*b*^ TND, target not detected.

^*c*^ SD, standard deviation.

### Accuracy of cobas SARS-CoV-2 Duo

We used the WHO International Standard for SARS-CoV-2 (NIBSC code 20/146) to verify the accuracy of the cobas SARS-CoV-2 Duo assay. The result of the standard was 7.66 log_10_ IU/mL, which was within the acceptance range of 7.70 ± 0.3 log_10_ IU/mL.

### Linearity of cobas SARS-CoV-2 Duo

**Fig 1:**
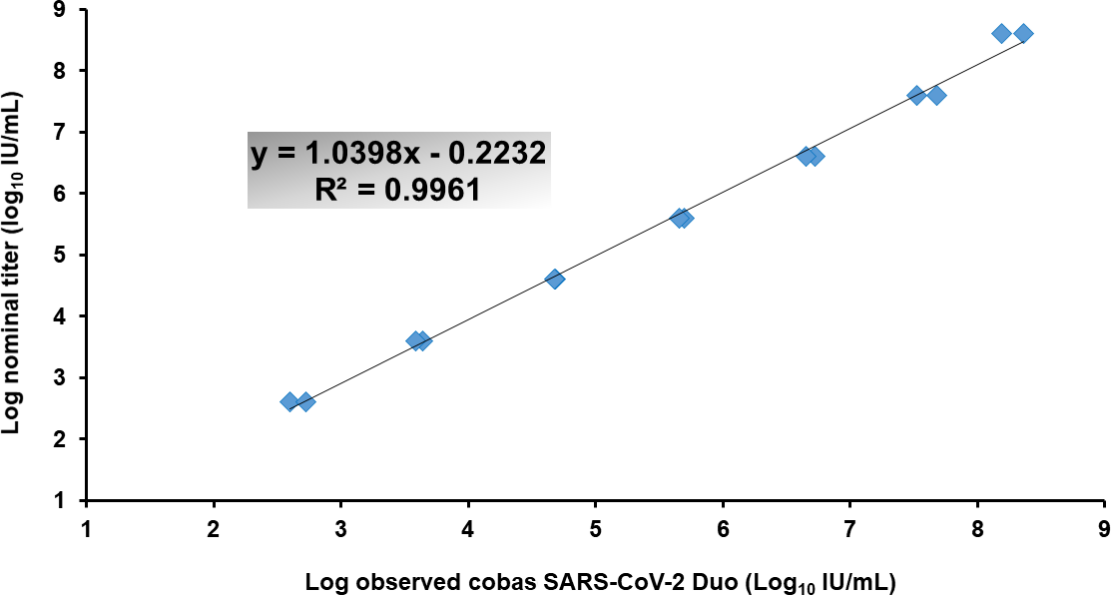
Linearity assessment of the cobas SARS-CoV-2 Duo.

Linearity of the SARS-CoV-2 Duo assay was assessed using pooled high viral load patient samples with 10-fold serial dilution, which was distributed among the AMR as much as possible, close to the upper limit of quantification (ULOQ) and the lower limit of quantification (LLOQ). The duplicated data in each titer level from the SARS-CoV-2 Duo assay were 8 log_10_: 8.36 and 8.19; 7 log_10_: 7.68 and 7.52; 6 log_10_: 6.73 and 6.66; 5 log_10_: 5.70 and 5.66, 4 log_10_: 4.68 and 4.68, 3 log_10_: 3.64 and 3.59; 2 log_10_: 2.60 and 2.72; one log_10_: both <LLOQ; 0 log_10_: target not detected and <LLOQ (Fig. 1). Overall, the *R* ^2^ was 0.9961 and, thus, acceptable based on the verification criterion.

### The agreement of cobas SARS-CoV-2 Duo and cobas SARS-CoV-2

A total of 201 clinical specimens were analyzed by the cobas SARS-CoV-2 Duo and the cobas SARS-CoV-2 assays. Forty-four specimens tested negative, and 157 specimens tested positive using the cobas SARS-CoV-2 analysis. On the same day, the cobas SARS-CoV-2 Duo quantitative reagent was used for testing. The outcomes included 33 negative results and 168 positive results. The 13 specimens which tested negative using the cobas SARS-CoV-2 tested positive via the cobas SARS-CoV-2 Duo; the viral load was <100 IU/mL in 12 specimens, and one specimen revealed a viral load of 2 log_10_ IU/mL. Overall, the positive agreement between the two assays was 98.7.5% (155 of 157); the negative agreement was 70.5% (31 of 44); and the total agreement was (186 of 201) 92.5% (Table 3).

**TABLE 3:** The agreement of cobas SARS-CoV-2 Duo and cobas SARS-CoV-2

SARS-CoV-2 Duo		SARS-CoV-2 genes				Agreement (%)
	*orf*1a and *orf*1ab	*orf*1ab/E (P/P)^*a*^	*orf*1ab/E (P/N)^*a*^ ^*, b*^	*orf*1ab/E (N/P)^*a*^ ^*,*^ ^*b*^	*orf*1ab/E (N/N)^*b*^	
TND^*c*^	33	0	0	2	31	93.9
<100 IU/mL	28	4	2	10	12	57.1
1.01E + 02~8.35E + 02	24	18	2	3	1	95.8
1.13E + 03~8.23E + 03	13	13	0	0	0	100
1.06E + 04 ~ 9.18E + 04	22	22	0	0	0	100
1.07E + 05 ~ 8.10E + 05	20	20	0	0	0	100
1.00E + 06 ~ 8.07E + 06	18	18	0	0	0	100
1.48E + 07 ~ 9.94E + 07	22	22	0	0	0	100
1.04E + 08 ~ 9.37E + 08	21	21	0	0	0	100
Total number	201	138	4	15	44	92.5
Total negative number	33				44	75.0
Total positive number	168	138	4	15		93.5

^*a*^ P, positive.

^*b*^ N, negative.

^*c*^ TND, target not detected.

### The correlation between viral load and Ct values between cobas SARS-CoV-2 Duo and cobas SARS-CoV-2

**Fig 2:**
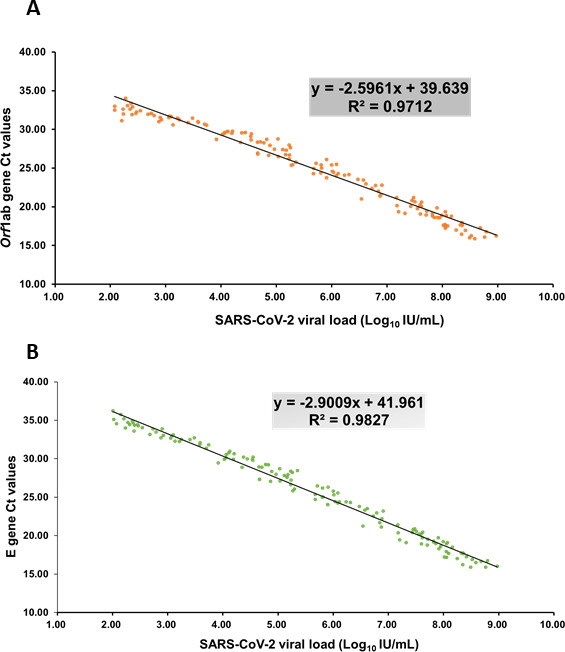
The correlation between the viral loads and Ct values of the cobas SARS-CoV-2 Duo and (**A**) the cobas SARS-CoV-2 orf1ab target and (**B**) the cobas SARS-CoV-2 E target.

The Ct values of the cobas SARS-CoV-2 Duo assays ranged from 13 to 44, while the Ct values of the cobas SARS-CoV-2 assays ranged from 15 to 37. The results indicated that the cobas SARS-CoV-2 Duo had a higher resolution in the Ct interpretation than the cobas SARS-CoV-2 (Table 4). The correlation between the *orf1ab* target Ct values and the viral loads in the cobas SARS-CoV-2 Duo equated to a *R* ^2^ of 0.9712 (Fig. 2A). The correlation of the E target Ct values and viral loads in cobas SARS-CoV-2 Duo was a *R* ^2^ of 0.9827 (Fig. 2B). These results indicated that the cobas SARS-CoV-2 had a good agreement with the cobas SARS-CoV-2 Duo, regarding data interpretation consistency and the Ct values compared to the viral loads (Table 4; Fig. 2).

**TABLE 4:** The correlation of viral load and Ct values between cobas SARS-CoV-2 Duo and cobas SARS-CoV-2

Viral load (IU/mL)	No.	cobas SARS-CoV-2 Duo		cobas SARS-CoV-2			
		orf1a and orf1ab duo targets		orf1ab gene		E gene	
		Ct values	Mean Ct values	Ct values (no. of specimens)	Mean Ct values	Ct values (no. of specimens)	Mean Ct values
TND^*a*^	33	NaN^*b*^	NaN^*b*^	NaN^*b*^ (33)	NaN^*b*^	35.41, 36.79, NaN^*b*^ (28)	36.10
<100	28	36.50 ~ 44.62	39.36	28.79 ~ 35.35	32.97	34.52 ~ 37.85	36.31
1.01E + 02 ~ 8.35E + 02	24	33.03 ~ 37.31	35.51	31.01 ~ 34.03, NaN^*b*^ (4)	32.28	32.72 ~ 36.23, NaN^*b*^ (3)	34.31
1.13E + 03 ~ 8.23E + 03	13	29.06 ~ 33.34	31.77	28.74 ~ 31.70	31.46	29.45 ~ 33.13	32.13
1.06E + 04 ~ 9.18E + 04	22	25.35 ~ 30.14	28.01	26.67 ~ 29.76	28.70	27.06 ~ 30.97	29.38
1.07E + 05 ~ 8.10E + 05	20	21.77 ~ 27.97	24.93	23.78 ~ 28.76	26.70	24.02 ~ 28.72	26.66
1.00E + 06 ~ 8.07E + 06	18	18.94 ~ 23.88	21.48	21.04 ~ 25.49	23.36	21.13 ~ 25.80	23.56
1.48E + 07 ~ 9.94E + 07	22	15.67 ~ 19.25	17.32	18.56 ~ 21.18	19.70	18.31 ~ 21.40	19.73
1.04E + 08 ~ 9.37E + 08	21	13.55 ~ 16.22	14.84	15.88 ~ 19.39	17.30	15.90 ~ 19.09	17.27

^*a*^ TND, target not detected.

^*b*^ NaN, not a number.

### The distribution of SARS-CoV-2 target Ct values in cobas SARS-CoV-2 Duo assay, cobas SRAS-CoV-2 assay, and cobas Liat assay

A total of 201 clinical SARS-CoV-2 positive specimens were analyzed using the cobas SARS-CoV-2 Duo, cobas SARS-CoV-2, and cobas Liat assays (Table 5). Each specimen was tested blinded using the three assays on the same day. Among these specimens, two tested positive using the cobas Liat assay but tested negative using the cobas SARS-CoV-2 Duo and cobas SRAS-CoV-2 assays. Considering the range of viral load <LLOQ to 2 log_10_, which was defined as near the detection limit, some inconsistencies among these three assays were observed. If the viral load was higher than three log_10_, the resulting agreement would be 100%. When the viral load was above ULOQ (>9 log_10_), the cobas SARS-CoV-2 Duo measured a Ct value of 10, the cobas SARS-CoV-2 a Ct value of 13, and the cobas Liat a Ct value of 9.

**TABLE 5:** The distribution of SARS-CoV-2 target Ct values in cobas SARS-CoV-2 Duo assay, cobas SRAS-CoV-2 assay, and Liat SARS-CoV-2 and influenza A/B (cobas Liat) assay

Viral load (IU/mL)	Viral load (log_10_ IU/mL)	No.	Cobas SARS-CoV-2 Duo		Cobas SARS-CoV-2				Cobas Liat	
			*orf*1a and *orf*1ab duo targets		*orf*1ab gene		E gene		orf1ab and N Duo targets	
			Ct values	Mean Ct values	Ct values	Mean C values	Ct values	Mean Ct values	Ct values	Mean Ct values
TND^*a*^	TND^*a*^	2	All negative	N/A^*b*^	All negative	N/A^*b*^	All negative	N/A^*b*^	32.82/33.81	33.31
<1.00E + 02	<2.00	10	36.79 ~ 40.63	38.72	All negative	N/A^*b*^	35.29 ~ 41.00 (5 positive)	38.15	31.05 ~ 32.58	31.85
1.12E + 02 ~ 7.65E + 02	2.05 ~ 2.88	20	33.54 ~ 37.62	35.30	31.21 ~ 33.99 (17 positive)	32.22	33.21 ~ 36.36	34.78	26.83 ~ 31.33	29.16
1.09E + 03 ~ 9.29E + 03	3.04 ~ 3.97	24	29.30 ~ 33.75	31.98	29.61 ~ 31.70	30.96	30.35 ~ 33.74	32.81	24.27 ~ 28.76	26.25
1.04E + 04 ~ 9.83E + 04	4.02~4.99	28	24.81 ~ 30.42	28.00	26.02 ~ 30.51	28.88	26.81 ~ 31.69	29.83	20.69 ~ 26.65	23.55
1.13E + 05 ~ 9.57E + 05	5.05~5.98	29	22.12 ~ 27.31	25.03	24.19 ~ 28.51	26.77	24.51 ~ 29.57	27.46	18.40 ~ 22.74	20.74
1.11E + 06 ~ 9.20E + 06	6.05~6.96	36	19.26 ~ 25.11	21.68	21.61 ~ 27.07	23.92	21.97 ~ 27.43	24.37	15.14 ~ 21.90	17.96
1.04E + 07 ~ 8.68E + 07	7.02 ~ 7.94	33	15.66 ~ 20.89	17.79	18.10 ~ 23.47	20.44	18.49 ~ 24.15	20.77	11.71 ~ 18.52	14.45
1.16E + 08 ~ 7.35E + 08	8.06 ~ 8.87	14	12.76 ~ 16.54	14.75	15.68 ~ 20.08	17.81	16.03 ~ 20.31	18.05	10.51 ~ 14.18	12.13
>1.00E + 09	>9.00	5	10.51 ~ 12.14	11.43	13.58 ~ 15.70	14.69	13.62 ~ 16.02	14.98	9.90 ~ 10.99	10.38

^*a*^ TND, target not detected.

^*b*^ N/A, not available.

### △Ct values of the SARS-CoV-2 targets in the three cobas assays

In this study, cobas SARS-CoV-2 can directly quantify the viral load, but cobas SRAS-CoV-2 assay and cobas Liat assay cannot. To help evaluate whether there is a certain linear relationship between the Ct values of different methods, we calculated △Ct by subtracting the Ct values obtained from the other two methods from the Ct value of the cobas SARS-CoV-2 Duo test. The △Ct trend between the SARS-CoV-2 Duo and SARS-CoV-2 *orf*1ab showed that the △Ct was negative at low concentration (log 2~3), then the trend reversed to positive from log 4, and the △Ct demonstrated a positive correlation with the concentration (i.e., as the viral load based on cobas SARS-CoV-2 increased, the △Ct increased) (Table 6). The trend phenomenon was the same using the SARS-CoV-2 E target. On the contrary, The △Ct trend between the SARS-CoV-2 Duo and SARS-CoV-2 *orf*1ab had a negative correlation regardless of viral load concentration (Table 6).

**TABLE 6:** △Ct values of SARS-CoV-2 targets in the three cobas assays

Viral load (IU/mL)	Viral load (log 10 IU/mL)	No.	Cobas SARS-CoV-2 duo		Cobas SARS-CoV-2				Cobas Liat	
			orf1a and orf1ab duo targets		orf1ab gene		E gene		orf1ab and N duo targets	
			Mean Ct values	△Ct values	Mean Ct values	△Ct values	Mean Ct values	△Ct values	Mean Ct values	△Ct values
TND	TND^*a*^	2	N/A	N/A	N/A	N/A	N/A	N/A	33.31	N/A
<1.00E + 02	<2.00	10	38.72	0	N/A	N/A	38.15	0.57	31.85	6.87
1.12E + 02 ~ 7.65E + 02	2.05~2.88	20	35.30	0	32.22	3.08	34.78	0.52	29.16	6.14
1.23E + 03 ~ 9.29E + 03	3.04~3.97	24	31.98	0	30.96	1.02	32.81	0.83	26.25	5.73
1.42E + 04 ~ 9.83E + 04	4.02~4.99	28	28.00	0	28.88	0.88	29.83	1.83	23.55	4.45
1.13E + 05 ~ 6.17E + 05	5.05~5.98	29	25.03	0	26.77	1.74	27.46	2.43	20.74	4.29
1.19E + 06 ~ 8.88E + 06	6.05~6.96	36	21.68	0	23.92	2.24	24.37	2.69	17.96	3.72
1.04E + 07 ~ 7.95E + 07	7.02~7.94	33	17.79	0	20.44	2.65	20.77	2.98	14.45	3.34
1.40E + 08 ~ 6.26E + 08	8.06~8.87	14	14.75	0	17.81	3.06	18.05	3.30	12.13	2.62
>1.00E + 09	>9.00	5	11.43	0	14.69	3.26	14.98	3.55	10.38	1.05

^*a*^ TND, target not detected.

### The correlation between Ct values and viral loads in the cobas SARS-CoV-2 and cobas Liat cobas SARS-CoV-2 and Inf A/B assays

**Fig 3:**
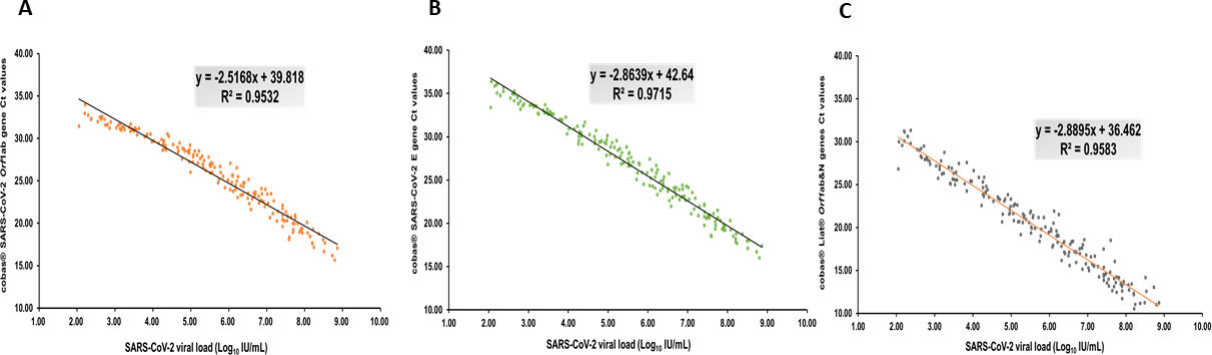
The correlation between the Ct values and viral loads in the cobas SARS-CoV-2 and cobas Liat cobas SARS-CoV-2 and Inf A/B assays. (**A**) The cobas SARS-CoV-2 orf1ab target. (**B**) The cobas SARS-CoV-2 E target. (**C**) The cobas Liat SARS-CoV-2 orf1ab and N target.

The correlation between the *orf*1ab target Ct values and viral loads in the cobas SARS-CoV-2 Duo was an *R* ^2^ of 0.9532. (Fig. 3A). The correlation between the E gene Ct values and viral loads in the cobas SARS-CoV-2 Duo was a *R* ^2^ of 0.9715 (Fig. 3B). The correlation of the orf1ab & N genes Ct values and viral loads in the cobas Liat assay was an *R* ^2^ of 0.9583 (Fig. 3C). These results demonstrated that the cobas SARS-CoV-2 and cobas Liat had good agreement with the cobas SARS-CoV-2 Duo in data interpretation consistency and Ct values compared to viral loads (Table 4).

## DISCUSSION

This study is the first to investigate the diagnostic performance of the cobas SARS-CoV-2 Duo assay. Most importantly, we demonstrated that the cobas SARS-CoV-2 Duo was a precise, accurate, and sensitive test according to the evidence as follows. First, the SD results of the low and high positive controls in the between-run and within-run were acceptable, which indicated that the cobas SARS-CoV-2 Duo was precise. Second, the accuracy and reliability of the cobas SARS-CoV-2 Duo were verified using the WHO International Standard for SARS-CoV-2. Third, the linearity of the cobas SARS-CoV-2 Duo was acceptable with an *R* ^2^ of 0.9961. Finally, there was a high positive agreement and total agreement between the cobas SARS-CoV-2 Duo and the two current tests, namely, the cobas SARS-CoV-2 and the cobas Liat assay. These findings confirmed the excellent performance of the cobas SARS-CoV-2 Duo as a quantitative test for SARS-CoV-2. Hence, our study supports the clinical use of this novel test.

In this study, we noted a negative agreement of only 75% between the cobas SARS-CoV-2 Duo and the two current assays (the cobas SARS-CoV-2 and the cobas Liat assay). The disagreement was due to the 13 clinical specimens that the cobas SARS-CoV-2 Duo tested positive, while the cobas SARS-CoV-2 tested negative. The viral load of these 13 clinical specimens was low based on the cobas SARS-CoV-2 Duo assay. In 12 of the 13 clinical specimens, the viral load was <100 IU/mL, and 1 specimen had a viral load of 2 log_10_ IU/mL. These results suggest that the cobas SARS-CoV-2 Duo may be more sensitive than the other two assays.

Consistent with the results of a previous report (24), we found that the Ct value was not a reliable tool for patient management or treatment guide. In this study, we observed that for a specific Ct value, the reported viral loads of the different assays could be significantly different. Therefore, an accurate viral load detection system, such as the SARS-CoV-2 Duo assay, is needed to provide accurate and consistent results to inform clinical management and further research. Fajnzylber et al. (28) reported that a higher prevalence of detectable SARS-CoV-2 viral load is associated with more severe respiratory diseases, lower absolute lymphocyte counts, and increased markers of inflammation (such as C-reactive protein and IL-6). Moreover, the SARS-CoV-2 viral load is associated with an increased risk of mortality. Therefore, further clinical studies on patients with a diverse range of COVID-19 disease severity, including those with mild disease and those with pneumonia, requiring hospitalization or intensive care, and individuals with resolved infection are needed to investigate the relationship between the SARS-CoV-2 viral loads obtained using the cobas SARS-CoV-2 Duo assay and the risk of disease progression.

In addition, the information on viral shedding is important in the containment of SARS-CoV-2 spread or transmission (29). In addition to symptom resolution, an objective assessment of viral load could help clinicians assess the virological eradication and treatment response. Even though patients may have symptoms that resolve but they still may be shedding virus particles and remain contagious (30, 31). Moreover, most of the patients who required hospitalization or resided in long-term care facilities were vulnerable to SARS-CoV-2. Therefore, the appropriate isolation/de-isolation of patients with COVID-19 to prevent nosocomial spread in these settings is critical. By acknowledging the viral load status, necessary quarantine or isolation may be implemented, especially within the healthcare system.

This study had several limitations. First, although SARS-CoV-2 can evolve rapidly, we did not assess the performance of cobas SARS-CoV-2 Duo specifically for each variant of concerns. In addition, the number of negative patient samples was relatively small, which may compromise the statistical reliability of negative predictive agreement or false positive rate. Second, we tested the diagnostic performance of cobas SARS-CoV-2 Duo, but we did not evaluate its associated other issues, such as easy-to-do protocol, alternate method, cost-effectiveness, bedside use, requirements of less infrastructure, or clinical impact. Third, this study compared cobas SARS-CoV-2 Duo with other assays of the same brand. To avoid potential bias, further studies comparing with other brands are warranted. Lastly, nasopharyngeal swab collections are variable and may not be the ideal specimen type for a quantitative assay. Although we found cobas SARS-CoV-2 Duo exhibited excellent diagnostic performance, which might provide useful information for management of patients with COVID-19, further study is needed to clarify the effect of different clinical sampling on its usefulness.

In conclusion, the diagnostic performance of the cobas SARS-CoV-2 Duo is appropriate. It is a precise, accurate, and sensitive method for the detection of SARS-CoV-2 RNA and is comparable to two qualitative assays (the cobas SARS-CoV-2 and the Liat cobas SARS-CoV-2 and Inf A/B). In contrast, Ct value could differ according to different assays, which suggests it is not reliable to use the Ct value to estimate viral load. Therefore, we need to utilize a quantitative detection test, such as the cobas SARS-CoV-2 Duo, to accurately measure the viral load.
